# 4,4′,5,5′-Tetra­phenyl-3,3′-[methyl­idene­bis(sulfanedi­yl)]bis­(4*H*-1,2,4-triazole)

**DOI:** 10.1107/S1600536810040729

**Published:** 2010-10-20

**Authors:** Bing Zhao, Zhuo Liu, Yan Gao, Bo Song, Qi-gang Deng

**Affiliations:** a Chemistry and Chemical Engineering Institute, Qiqihar University, Heilongjiang Qiqihar 161006, People’s Republic of China; b School of Chemical Engineering, University of Science and Technology LiaoNing, Anshan 114051, People’s Republic of China

## Abstract

The asymmetric unit of the title compound, C_29_H_22_N_6_S_2_, contains one half-mol­ecule situated on a twofold rotational axis. The two triazole rings form a dihedral angle of 27.6 (2)°. In the crystal, weak inter­molecular C—H⋯N hydrogen bonds link the mol­ecules into ribbons extending along [001].

## Related literature

For related structures, see: Özel Güven *et al.* (2008**a* ▶,b* ▶). For the pharmacological properties of triazole derivatives, see: Paulvannan *et al.* (2001 ▶); Wahbi *et al.* (1995 ▶).
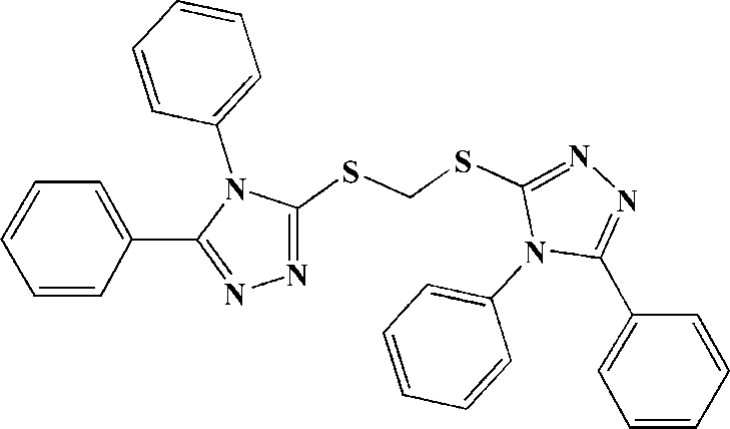


## Experimental

### 

#### Crystal data

C_29_H_22_N_6_S_2_
*M* *_r_* = 518.65
Orthorhombic, 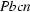

*a* = 30.449 (6) Å
*b* = 8.2759 (17) Å
*c* = 9.7353 (19) Å
*V* = 2453.2 (9) Å^3^
*Z* = 4
Mo *K*α radiation
μ = 0.25 mm^−1^
*T* = 113 K
0.22 × 0.18 × 0.12 mm

#### Data collection

Rigaku Saturn CCD area-detector diffractometer
Absorption correction: multi-scan (*CrystalClear*; Rigaku/MSC, 1999 ▶) *T*_min_ = 0.947, *T*_max_ = 0.971
17244 measured reflections
2147 independent reflections
1970 reflections with *I* > 2σ(*I*)
*R*_int_ = 0.045

#### Refinement

*R*[*F* ^2^ > 2σ(*F* ^2^)] = 0.040
*wR*(*F* ^2^) = 0.125
*S* = 1.10
2147 reflections
169 parameters
H-atom parameters constrained
Δρ_max_ = 0.39 e Å^−3^
Δρ_min_ = −0.29 e Å^−3^

### 

Data collection: *CrystalClear* (Rigaku/MSC, 1999 ▶); cell refinement: *CrystalClear*; data reduction: *CrystalClear*; program(s) used to solve structure: *SHELXS97* (Sheldrick, 2008 ▶); program(s) used to refine structure: *SHELXL97* (Sheldrick, 2008 ▶); molecular graphics: *SHELXTL* (Sheldrick, 2008 ▶); software used to prepare material for publication: *SHELXTL*.

## Supplementary Material

Crystal structure: contains datablocks global, I. DOI: 10.1107/S1600536810040729/cv2773sup1.cif

Structure factors: contains datablocks I. DOI: 10.1107/S1600536810040729/cv2773Isup2.hkl

Additional supplementary materials:  crystallographic information; 3D view; checkCIF report

## Figures and Tables

**Table 1:** Hydrogen-bond geometry (Å, °)

*D*—H⋯*A*	*D*—H	H⋯*A*	*D*⋯*A*	*D*—H⋯*A*
C12—H12⋯N2^i^	0.95	2.49	3.438 (2)	173

Symmetry code: (i) 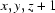.

**Fig. 1.**
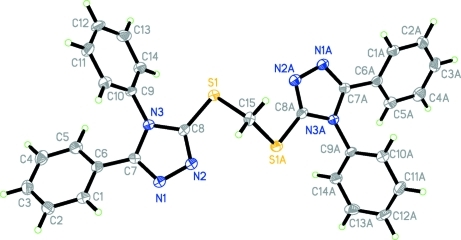
View of (I) showing the atom-labelling scheme [symmetry code: (A) -x + 1,y,-z + 1/2]. Displacement ellipsoids are drawn at the 50% probability level.

## supplementary crystallographic information

### Comment

Functionalized 1,2,4-triazole derivatives received considerable attention due to their antihypertensive, antifungal and antibacterial properties (Paulvannan *et al.*, 2001; Wahbi *et al.*, 1995). Some crystal structures of 1*H*-1,2,4-triazole ring containing ether derivatives have been reported recently (Özel Güven *et al.*, 2008*a*,*b*). Herewith we report the synthesis and crystal structure of the title compound, (I).

In (I) (Fig.1),  the molecule is situated on a twofold rotational axis, so asymmetric unit contains a half of the molecule. The 1,2,4-triazole ring is planar with an r.m.s. deviation of 0.023 (2)Å and maximum deviation of 0.0037 (2)Å for atoms C8. The C8 atom shows a distorted C*_sp_*^2^ hybridization state with the bond angles of 110.98 (16)° (N2—C8—N3) and 126.50 (14)°(N2—C8—S1). The triazole ring and two attached phenyl rings (C1—C6 and C9—C14) form dihedral angles of 34.3 (2) and 62.2 (2)°, respectively. The dihedral angle between the two phenyl rings is 61.2 (2)°. As a result of π-π conjugation, the C*_sp_*^2^-S bond length [S1—C8 = 1.7439 (2) Å] is significantly shorter than the C*_sp_*^3^-S bond length [S1—C15 = 1.8092 (2) Å].

In the crystal structure, weak intermolecular C—H···N hydrogen bonds (Table 1) link the molecules into ribbons extended in direction [001].

### Experimental

A suspension of 4,5-diphenyl-4*H*-1,2,4-triazole-3-thiol (2.0 mmol) and 1,1-dibromomethane (1.0 mmol) in ethanol (10 ml) was stirred at room temperature. The reaction progress was monitored *via* TLC. The resulting precipitate was filtered off, washed with cold ethanol, dried and purified to give the target product as light yellow solid in 95% yield. Crystals of (I) suitable for single-crystal X-ray analysis were grown by slow evaporation of a solution in chloroform-ethanol (1:1).

### Refinement

All H atoms were positioned geometrically and refined as riding (C—H = 0.95–0.99 Å) and allowed to ride on their parent atoms, with *U*_iso_(H) = 1.2*U*_eq_ (C).

### Crystal data

**Table:**

C_29_H_22_N_6_S_2_	*F*(000) = 1080
*M**_r_* = 518.65	*D*_x_ = 1.404 Mg m^−^^3^
Orthorhombic, *P**b**c**n*	Mo *K*α radiation, λ = 0.71073 Å
Hall symbol: -P 2n 2ab	Cell parameters from 6468 reflections
*a* = 30.449 (6) Å	θ = 1.3–27.9°
*b* = 8.2759 (17) Å	µ = 0.25 mm^−^^1^
*c* = 9.7353 (19) Å	*T* = 113 K
*V* = 2453.2 (9) Å^3^	Prism, colorless
*Z* = 4	0.22 × 0.18 × 0.12 mm

### Data collection

**Table:**

Rigaku Saturn CCD area-detector diffractometer	2147 independent reflections
Radiation source: rotating anode	1970 reflections with *I* > 2σ(*I*)
confocal	*R*_int_ = 0.045
Detector resolution: 7.31 pixels mm^-1^	θ_max_ = 25.0°, θ_min_ = 1.3°
φ and ω scans	*h* = −36→36
Absorption correction: multi-scan (*CrystalClear*; Rigaku/MSC, 1999)	*k* = −9→9
*T*_min_ = 0.947, *T*_max_ = 0.971	*l* = −11→10
17244 measured reflections	

### Refinement

**Table:**

Refinement on *F*^2^	Secondary atom site location: difference Fourier map
Least-squares matrix: full	Hydrogen site location: inferred from neighbouring sites
*R*[*F*^2^ > 2σ(*F*^2^)] = 0.040	H-atom parameters constrained
*wR*(*F*^2^) = 0.125	*w* = 1/[σ^2^(*F*_o_^2^) + (0.0826*P*)^2^ + 0.5471*P*] where *P* = (*F*_o_^2^ + 2*F*_c_^2^)/3
*S* = 1.10	(Δ/σ)_max_ = 0.002
2147 reflections	Δρ_max_ = 0.39 e Å^−^^3^
169 parameters	Δρ_min_ = −0.29 e Å^−^^3^
0 restraints	Extinction correction: *SHELXL97* (Sheldrick, 2008), Fc^*^=kFc[1+0.001xFc^2^λ^3^/sin(2θ)]^-1/4^
Primary atom site location: structure-invariant direct methods	Extinction coefficient: 0.050 (4)

### Special details

**Table:**

Geometry. All e.s.d.'s (except the e.s.d. in the dihedral angle between two l.s. planes) are estimated using the full covariance matrix. The cell e.s.d.'s are taken into account individually in the estimation of e.s.d.'s in distances, angles and torsion angles; correlations between e.s.d.'s in cell parameters are only used when they are defined by crystal symmetry. An approximate (isotropic) treatment of cell e.s.d.'s is used for estimating e.s.d.'s involving l.s. planes.
Refinement. Refinement of *F*^2^ against ALL reflections. The weighted *R*-factor *wR* and goodness of fit *S* are based on *F*^2^, conventional *R*-factors *R* are based on *F*, with *F* set to zero for negative *F*^2^. The threshold expression of *F*^2^ > σ(*F*^2^) is used only for calculating *R*-factors(gt) *etc*. and is not relevant to the choice of reflections for refinement. *R*-factors based on *F*^2^ are statistically about twice as large as those based on *F*, and *R*- factors based on ALL data will be even larger.

### Fractional atomic coordinates and isotropic or equivalent isotropic displacement parameters (Å^2^)

**Table:**

	*x*	*y*	*z*	*U*_iso_*/*U*_eq_	Occ. (<1)
S1	0.484576 (14)	0.18721 (6)	0.40035 (4)	0.0246 (2)	
N1	0.37084 (5)	0.2686 (2)	0.23508 (15)	0.0243 (4)	
N2	0.41528 (5)	0.23130 (19)	0.22557 (15)	0.0244 (4)	
N3	0.39880 (5)	0.27134 (18)	0.44399 (15)	0.0185 (4)	
C1	0.28189 (6)	0.2592 (2)	0.34631 (19)	0.0254 (4)	
H1	0.2872	0.1913	0.2694	0.030*	
C2	0.23919 (6)	0.2897 (3)	0.3878 (2)	0.0288 (5)	
H2	0.2153	0.2438	0.3388	0.035*	
C3	0.23142 (6)	0.3863 (3)	0.49991 (19)	0.0299 (5)	
H3	0.2021	0.4061	0.5291	0.036*	
C4	0.26622 (7)	0.4548 (2)	0.5704 (2)	0.0302 (5)	
H4	0.2606	0.5211	0.6481	0.036*	
C5	0.30922 (6)	0.4281 (2)	0.52912 (18)	0.0260 (4)	
H5	0.3329	0.4775	0.5768	0.031*	
C6	0.31729 (6)	0.3280 (2)	0.41710 (18)	0.0207 (4)	
C7	0.36147 (6)	0.2918 (2)	0.36533 (19)	0.0203 (4)	
C8	0.43093 (6)	0.2328 (2)	0.35103 (18)	0.0207 (4)	
C9	0.40330 (6)	0.2658 (2)	0.59099 (17)	0.0190 (4)	
C10	0.37948 (6)	0.1537 (2)	0.66540 (18)	0.0251 (4)	
H10	0.3601	0.0812	0.6200	0.030*	
C11	0.38411 (6)	0.1481 (3)	0.80686 (19)	0.0291 (5)	
H11	0.3673	0.0734	0.8590	0.035*	
C12	0.41327 (7)	0.2513 (3)	0.87285 (19)	0.0298 (5)	
H12	0.4167	0.2464	0.9698	0.036*	
C13	0.43734 (6)	0.3613 (2)	0.79676 (19)	0.0297 (5)	
H13	0.4576	0.4310	0.8417	0.036*	
C14	0.43218 (6)	0.3708 (2)	0.65523 (18)	0.0240 (4)	
H14	0.4482	0.4482	0.6033	0.029*	
C15	0.5000	0.0719 (3)	0.2500	0.0245 (6)	
H15A	0.4751	0.0011	0.2250	0.029*	0.50
H15B	0.5249	0.0011	0.2750	0.029*	0.50

### Atomic displacement parameters (Å^2^)

**Table:**

	*U*^11^	*U*^22^	*U*^33^	*U*^12^	*U*^13^	*U*^23^
S1	0.0196 (3)	0.0371 (4)	0.0171 (3)	0.00410 (18)	−0.00078 (16)	−0.00138 (18)
N1	0.0225 (8)	0.0335 (9)	0.0168 (8)	0.0024 (6)	−0.0003 (6)	0.0006 (6)
N2	0.0226 (8)	0.0324 (9)	0.0181 (8)	0.0017 (7)	0.0001 (6)	0.0006 (6)
N3	0.0188 (8)	0.0224 (8)	0.0143 (8)	0.0001 (6)	−0.0011 (6)	0.0003 (6)
C1	0.0261 (10)	0.0308 (10)	0.0193 (10)	0.0008 (8)	−0.0022 (7)	0.0001 (8)
C2	0.0223 (10)	0.0385 (12)	0.0256 (11)	−0.0025 (8)	−0.0010 (7)	0.0084 (8)
C3	0.0244 (9)	0.0360 (12)	0.0292 (10)	0.0067 (8)	0.0054 (8)	0.0100 (9)
C4	0.0344 (10)	0.0294 (11)	0.0267 (10)	0.0083 (8)	0.0062 (8)	0.0002 (9)
C5	0.0286 (9)	0.0269 (10)	0.0224 (10)	0.0026 (8)	−0.0009 (8)	0.0000 (8)
C6	0.0221 (9)	0.0232 (10)	0.0169 (9)	0.0026 (7)	−0.0001 (7)	0.0045 (7)
C7	0.0201 (9)	0.0231 (9)	0.0176 (9)	0.0004 (7)	−0.0023 (7)	0.0008 (7)
C8	0.0223 (9)	0.0248 (9)	0.0152 (9)	0.0005 (7)	0.0010 (7)	0.0002 (7)
C9	0.0205 (9)	0.0240 (10)	0.0125 (9)	0.0044 (7)	−0.0008 (7)	0.0008 (7)
C10	0.0259 (9)	0.0293 (10)	0.0203 (10)	−0.0010 (8)	−0.0008 (7)	0.0007 (8)
C11	0.0305 (10)	0.0361 (11)	0.0207 (9)	0.0039 (9)	0.0058 (8)	0.0070 (8)
C12	0.0348 (11)	0.0417 (12)	0.0130 (9)	0.0129 (9)	−0.0014 (8)	−0.0001 (8)
C13	0.0317 (10)	0.0355 (11)	0.0218 (9)	0.0040 (9)	−0.0063 (8)	−0.0089 (8)
C14	0.0253 (9)	0.0265 (10)	0.0201 (9)	0.0007 (7)	−0.0014 (7)	−0.0014 (8)
C15	0.0228 (12)	0.0283 (14)	0.0224 (13)	0.000	0.0048 (10)	0.000

### Geometric parameters (Å, °)

**Table:**

S1—C8	1.7439 (19)	C5—C6	1.391 (3)
S1—C15	1.8092 (15)	C5—H5	0.9500
N1—C7	1.314 (2)	C6—C7	1.467 (3)
N1—N2	1.391 (2)	C9—C10	1.382 (3)
N2—C8	1.311 (2)	C9—C14	1.386 (3)
N3—C8	1.370 (2)	C10—C11	1.385 (3)
N3—C7	1.381 (2)	C10—H10	0.9500
N3—C9	1.438 (2)	C11—C12	1.389 (3)
C1—C2	1.385 (3)	C11—H11	0.9500
C1—C6	1.400 (3)	C12—C13	1.384 (3)
C1—H1	0.9500	C12—H12	0.9500
C2—C3	1.373 (3)	C13—C14	1.389 (3)
C2—H2	0.9500	C13—H13	0.9500
C3—C4	1.384 (3)	C14—H14	0.9500
C3—H3	0.9500	C15—S1^i^	1.8092 (15)
C4—C5	1.387 (3)	C15—H15A	0.9900
C4—H4	0.9500	C15—H15B	0.9900
			
C8—S1—C15	97.73 (6)	N2—C8—N3	110.98 (16)
C7—N1—N2	107.93 (14)	N2—C8—S1	126.50 (14)
C8—N2—N1	106.82 (14)	N3—C8—S1	122.50 (13)
C8—N3—C7	104.47 (15)	C10—C9—C14	121.16 (17)
C8—N3—C9	125.57 (15)	C10—C9—N3	119.54 (16)
C7—N3—C9	129.35 (15)	C14—C9—N3	119.29 (16)
C2—C1—C6	120.33 (19)	C9—C10—C11	119.34 (18)
C2—C1—H1	119.8	C9—C10—H10	120.3
C6—C1—H1	119.8	C11—C10—H10	120.3
C3—C2—C1	119.98 (19)	C10—C11—C12	120.29 (18)
C3—C2—H2	120.0	C10—C11—H11	119.9
C1—C2—H2	120.0	C12—C11—H11	119.9
C2—C3—C4	120.05 (18)	C13—C12—C11	119.70 (18)
C2—C3—H3	120.0	C13—C12—H12	120.2
C4—C3—H3	120.0	C11—C12—H12	120.2
C3—C4—C5	120.90 (19)	C12—C13—C14	120.54 (18)
C3—C4—H4	119.5	C12—C13—H13	119.7
C5—C4—H4	119.5	C14—C13—H13	119.7
C4—C5—C6	119.26 (18)	C9—C14—C13	118.94 (18)
C4—C5—H5	120.4	C9—C14—H14	120.5
C6—C5—H5	120.4	C13—C14—H14	120.5
C5—C6—C1	119.46 (17)	S1—C15—S1^i^	116.36 (15)
C5—C6—C7	123.54 (17)	S1—C15—H15A	108.2
C1—C6—C7	116.99 (17)	S1^i^—C15—H15A	108.2
N1—C7—N3	109.79 (16)	S1—C15—H15B	108.2
N1—C7—C6	124.08 (16)	S1^i^—C15—H15B	108.2
N3—C7—C6	126.09 (16)	H15A—C15—H15B	107.4
			
C7—N1—N2—C8	0.3 (2)	N1—N2—C8—S1	177.52 (14)
C6—C1—C2—C3	−0.7 (3)	C7—N3—C8—N2	0.7 (2)
C1—C2—C3—C4	0.8 (3)	C9—N3—C8—N2	172.43 (17)
C2—C3—C4—C5	0.3 (3)	C7—N3—C8—S1	−177.52 (13)
C3—C4—C5—C6	−1.4 (3)	C9—N3—C8—S1	−5.8 (3)
C4—C5—C6—C1	1.4 (3)	C15—S1—C8—N2	−20.82 (19)
C4—C5—C6—C7	−179.98 (17)	C15—S1—C8—N3	157.12 (16)
C2—C1—C6—C5	−0.4 (3)	C8—N3—C9—C10	−113.1 (2)
C2—C1—C6—C7	−179.05 (17)	C7—N3—C9—C10	56.5 (3)
N2—N1—C7—N3	0.2 (2)	C8—N3—C9—C14	65.6 (2)
N2—N1—C7—C6	−177.72 (17)	C7—N3—C9—C14	−124.8 (2)
C8—N3—C7—N1	−0.5 (2)	C14—C9—C10—C11	1.0 (3)
C9—N3—C7—N1	−171.81 (16)	N3—C9—C10—C11	179.62 (16)
C8—N3—C7—C6	177.31 (17)	C9—C10—C11—C12	−1.7 (3)
C9—N3—C7—C6	6.0 (3)	C10—C11—C12—C13	0.8 (3)
C5—C6—C7—N1	−146.00 (19)	C11—C12—C13—C14	0.8 (3)
C1—C6—C7—N1	32.6 (3)	C10—C9—C14—C13	0.6 (3)
C5—C6—C7—N3	36.5 (3)	N3—C9—C14—C13	−178.03 (16)
C1—C6—C7—N3	−144.92 (18)	C12—C13—C14—C9	−1.5 (3)
N1—N2—C8—N3	−0.6 (2)	C8—S1—C15—S1^i^	80.05 (6)

Symmetry codes: (i) −*x*+1, *y*, −*z*+1/2.

### Hydrogen-bond geometry (Å, °)

**Table:**

*D*—H···*A*	*D*—H	H···*A*	*D*···*A*	*D*—H···*A*
C12—H12···N2^ii^	0.95	2.49	3.438 (2)	173

Symmetry codes: (ii) *x*, *y*, *z*+1.
